# Draft genome sequence of *Shewanella algae* JC874

**DOI:** 10.1128/mra.00499-25

**Published:** 2025-07-28

**Authors:** Mahima Dhurka, Atham Hari Naga Papa Rao, Ria Biswas, Lakshmanan Vighnesh, Chintalapati Sasikala, Chintalapati Venkata Ramana

**Affiliations:** 1 Department of Plant Sciences, School of Life Sciences, University of Hyderabad 28614, Hyderabad, Telangana, India; 2 Smart Microbiology Services, Secunderabad, India; University of California Riverside, Riverside, California, USA

**Keywords:** *Shewanella algae*, predatory

## Abstract

*Shewanella algae* are ubiquitous. They are pathogens affecting aquatic organisms and are emerging as pathogens to humans. Here, we present the genome of *S. algae* strain JC874, isolated from an alga, *Codium* sp. It showed plaques on mixed culture streaking with *Bacillus tequilensis* JC1001. The genome size is 4.8 Mb.

## ANNOUNCEMENT

**Fig 1:**
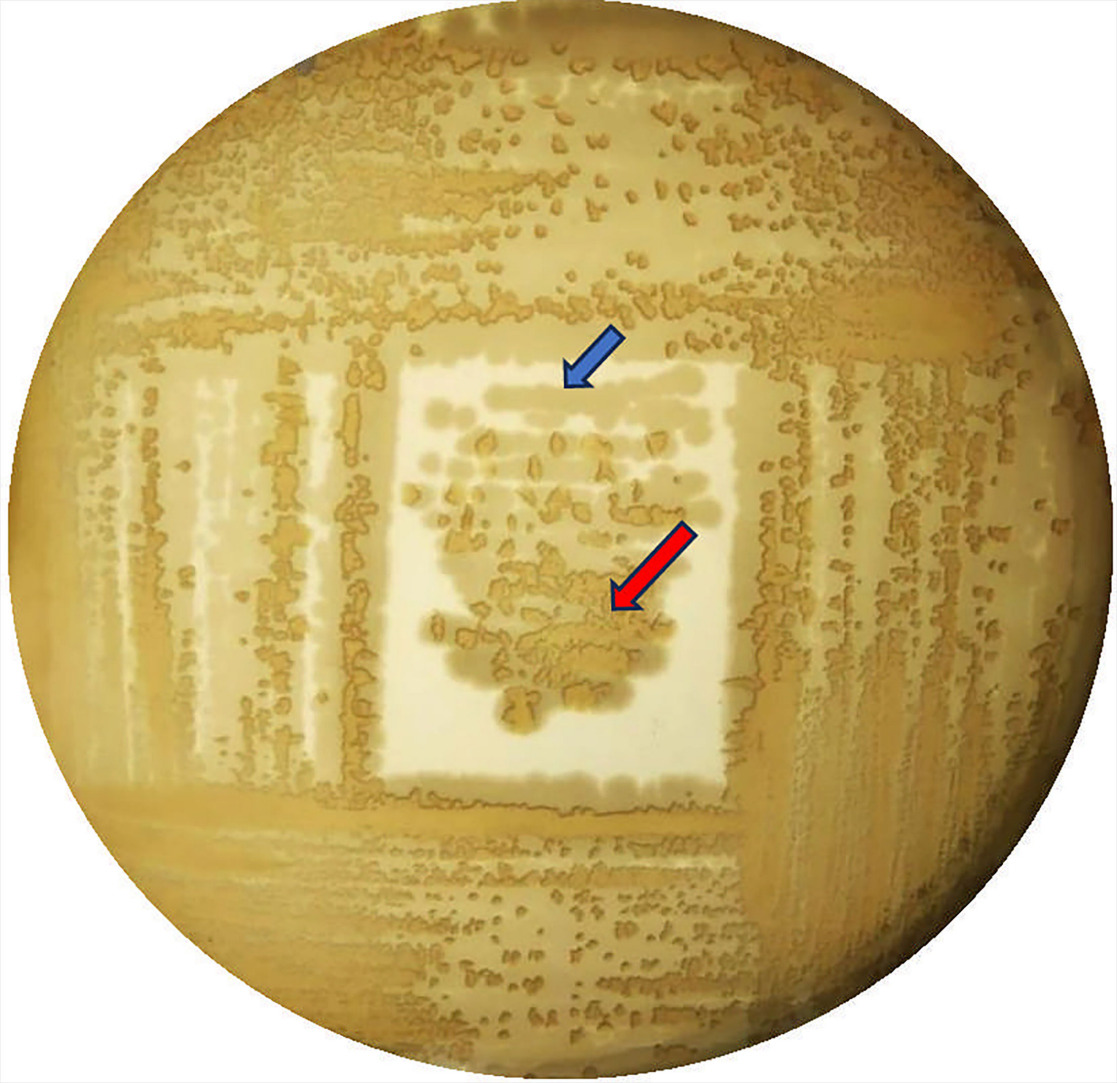
Plate showing the plaques formed by *Shewanella algae* JC874 on *B. tequilensis* JC1001. The blue arrow shows strain JC1001, and the red arrow shows the plaques. Both cultures did not show any plaques when cultured independently (data not shown).

There are reports that marine macroalgae have antibacterial properties (1). In this study, we examined the antimicrobial properties of *Codium* sp., a marine macroalga collected from Pudumadam (9.2771°N, 78.9939°E) of the Gulf of Mannar, Tamil Nadu, India. A small (1 cm × 1 cm) piece of the alga was placed on a lawn of *Bacillus tequilensis* JC1001 grown on Luria-broth (LB, HIMEDIA, M575) agar plate. After incubation at 37°C for 24 h, a zone of inhibition around the algal sample was observed. We further sub-cultured from the zone around the algal sample and carried out mixed culture streaking, on which we could observe plaques (Fig. 1), which resembled the plaques formed by bacteriophages (2) and predatory bacteria (3). Through subsequent streaking, we could isolate the axenic culture and assign it as strain JC874. The absence of plaques in axenic culture indicates that the bacteriophages are unlikely to be involved, suggesting that the strain JC874 may be a putative predator.

For identification of the strain JC874, its 16S rRNA gene was amplified using PCR with the primer pair 27F and 1492R. BLAST analysis of the gene sequence (1,399 bp) showed the highest identity of 99.3% with the type strain of *Shewanella algae* JCM21037^T^. A single colony of the strain JC874 was inoculated in LB broth (HIMEDIA, M575), cultured for about 24 h, and was outsourced to LifeCell International Pvt Ltd, where the genomic DNA was extracted using the DNeasy Blood and Tissue kit (QIAGEN, 69504). A genomic DNA library was prepared using the NEBNext Ultra DNA Library Preparation Kit (Illumina) and sequenced on the NovaSeq 6000 platform with 150 bp paired-end reads to generate raw sequencing data. A total of 34,331,398 raw reads were generated and processed using fastp v0.12.4 (4), which, after QC, gave 34,201,152 reads. The reads were assembled into contigs using Unicycler v0.5.0 (5), and contigs less than 200 bp were discarded. A total of 71 contigs were generated, resulting in a genome assembly size of 4.8 Mb with a N_50_ 305.5 kb. The statistics of the assembled genome were assessed using QUAST v5.0.2 (6), and read alignment of the assembly was checked by mapping back to the reads using Bowtie2 v2.4.5 (7), which showed an alignment of 98.7%. The default parameters were used for all software. The genome was submitted to NCBI and is available with the accession number JAWJDV000000000. The genome was annotated by the NCBI Prokaryotic Genome Annotation Pipeline (PGAP) version 6.6. The total genome encodes for 4,206 protein-coding genes, 3 rRNA, 95 tRNA, and a GC content of 53%. The genome shows a completeness of 99.46% and 0.04% contamination, according to PGAP. The genome of strain JC874 provides a valuable resource for exploring its putative predatory behavior and facilitates comparative analyses with other known bacterial predators.

## Data Availability

The genome sequence of *Shewanella algae* JC874 is available in GenBank under the BioProject PRJNA1023378, BioSample SAMN37651311, and genome accession number JAWJDV000000000. The raw reads were deposited in the Sequence Read Archive (SRA) database under the accession number SRR26361328. The 16S rRNA gene sequence is deposited in GenBank under the accession OR394137.
